# Intensity of Respiratory Cortical Arousals Is a Distinct Pathophysiologic Feature and Is Associated with Disease Severity in Obstructive Sleep Apnea Patients

**DOI:** 10.3390/brainsci11030282

**Published:** 2021-02-25

**Authors:** Katharina Bahr, Vincent Geisler, Tilman Huppertz, Sergiu Groppa, Christoph Matthias, Haralampos Gouveris, Muthuraman Muthuraman

**Affiliations:** 1 Sleep Medicine Center, Department of Otorhinolaryngology, Medical Center of the University of Mainz, 55131 Mainz, Germany; katharina.bahr@unimedizin-mainz.de (K.B.); vincentgeisler@yahoo.de (V.G.); Tilman.huppertz@unimedizin-mainz.de (T.H.); christoph.matthias@unimedizin-mainz.de (C.M.); Haralampos.Gouveris@unimedizin-mainz.de (H.G.); 2 Movement Disorders and Neurostimulation, Biomedical Statistics and Multimodal Signal Processing Unit, Department of Neurology, Medical Center of the University of Mainz, 55131 Mainz, Germany; segroppa@uni-mainz.de

**Keywords:** sleep apnea, sleep-disturbed breathing, respiratory, arousal, microstructure

## Abstract

Background: We investigated whether the number, duration and intensity of respiratory arousals (RA) on C3-electroencephalographic (EEG) recordings correlate with polysomnography (PSG)-related disease severity in obstructive sleep apnea (OSA) patients. We also investigated if every patient might have an individual RA microstructure pattern, independent from OSA-severity. Methods: PSG recordings of 20 OSA patients (9 female; age 27–80 years) were analyzed retrospectively. Correlation coefficients were calculated between RA microstructure (duration, EEG-intensity) and RA number and respiratory disturbance index (RDI), oxygen desaturation index (ODI) and arousal index (AI). Intraclass correlations (ICC) for both RA duration and intensity were calculated. Sleep stage-specific and apnea- and hypopnea-specific analyses were also done. The probability distributions of duration and intensity were plotted, interpolated with a kernel which fits the distribution. A Bayesian posterior distribution analysis and pair-wise comparisons of each patient with all other 19 patients were performed. Results: Of the analyzed 2600 RA, strong positive correlations were found between average RA intensity and both RDI and AI. The number of PSG-recorded RA was strongly positively correlated with RDI. Significant correlations between average RA intensity in REM, NREM2 and NREM3 sleep stages and total ODI were identified. No sleep stage-specific correlations of arousal microstructure with age, sex, RDI or AI were identified. Although between-subjects ICC values were <0.25, within-subject ICC values were all >0.7 (all *p* < 0.05). While apnea-related RA duration did not differ from hypopnea-related RA duration, RA intensity was significantly higher (*p* = 0.00135) in hypopneas than in apneas. A clear individual pattern of arousal duration for each patient was made distinct. For arousal intensity, a Gaussian distribution was identified in most patients. The Bayesian statistics regarding the arousal microstructure showed significant differences between each pair of patients. Conclusions: Each individual patient with OSA might have an individual pattern of RA intensity and duration indicating a distinct individual pathophysiological feature. Arousal intensity was significantly higher in hypopneic than in apneic events and may be related causally to the diminished (compared to apneas) respiratory distress associated with hypopneas. RA intensity in REM, NREM2 and NREM3 strongly correlated with ODI.

## 1. Introduction

Obstructive sleep apnea (OSA) is characterized by intermittent narrowing and collapse of the pharyngeal airway during sleep. These interruptions of breathing cause blood-gas disturbances and disrupt the sleep continuity as they are associated with the arousals [1]. During sleep, physiologic, movement-related and respiratory arousals may occur. In contrast to movement-related and physiologic arousals, respiratory arousals (RA) are directly related to sleep-disordered breathing (SDB). Frequency and intensity of arousals seem to have different effects on sleep and breathing [2]. The exact mechanisms of the development of RA have not yet been identified and are the subject of intensive investigation. It appears that different factors that increase the breathing effort, e.g., hypercapnia and hypoxia, as well as mechanical respiratory events during sleep, trigger a respiratory arousal [3].

According to the American Academy of Sleep Medicine (AASM) scoring rules, an arousal is scored as an all-or-none event. It is defined as an abrupt shift of the EEG frequency including alpha, theta and/or frequencies greater than 16 Hz (but not spindles) that lasts at least 3 s, with at least 10 s of stable sleep preceding the change [4]. The phenotype of an arousal is very variable. It can either occur during inspiration or expiration [5]. Arousals usually vary in duration and intensity (amplitude) [6,7].

Additionally, RAs seem to have an ambivalent role in patients with OSA. On the one hand, it is a common presumption that RAs suppress life-threatening apneic events during sleep. The obvious connection between an obstructive event and an RA has been believed to be the essential part of the pathogenesis of a RA; especially since Remmers et al. proclaimed that a RA is required for the airway to open in cases of obstructive respiratory events during sleep [8]. Nowadays, we know after more extensive study of RAs in OSA patients that RAs may be unnecessary in OSA patients. It was observed that in 10–25% of obstructive events an arousal may not be observed at the time of upper airway opening [6,9,10,11]. One study proclaimed that in 40% of the patients the upper airway may open during an obstructive event without any associated RA [6]. In total, 75% of the patients experienced obstructive events either without an arousal or with an arousal which succeeded the airway opening [6]. It has therefore been suggested that stimuli that lead to arousals and stimuli that are needed to activate the upper airway dilator muscles may be the same; thus, arousals may appear to succeed an obstructive event [1]. In many of these cases, a loud snort associated with upper airway opening precedes the actual arousal, and in these cases, it is possible that the arousal might be noise- rather than obstruction-related [6].

Different chemical factors are also believed to act as stimuli for triggering RAs when reaching a certain threshold value, e.g., blood oxygen pressure, CO-2 partial pressure or oxygen saturation [12]. In particular, oxygen desaturation seems to play an important role in the genesis of RAs, as studies in OSA patients have shown that the maximum desaturations of SaO_2_ during respiratory events with arousals are larger than the desaturations in events without arousals [13].

Another possible association in the process of arousals seems to indicate a link between cerebrospinal fluid (CSF) flow and EEG-activity. Indeed, neural slow waves are followed by hemodynamic oscillations, which are coupled with CSF flow [14].

Additionally, the sleep stages seem to play an important role in the genesis and process of RAs. Clinical data have shown that respiratory events as well as respiratory arousals (RA) are most prevalent in lighter stages of non-rapid eye movement (NREM) sleep [15,16]. The respiratory arousal, not being an essential part of airway opening, needs further investigation. Apart from the sleep stages, studies have shown that the amount of stimuli that lead to an arousal seem to differ not only among patients [17,18] but also regarding the same individual during the night [19,20], whereas the number of stimuli that leads to an opening of the upper airway might differ among individuals; however, it seems to be constant regarding any given patient during sleep [21,22].

The total number of arousals recorded during an overnight polysommographic (PSG) recording is closely related to the respiratory disturbance index (RDI). Patients with a high RDI are proven to have a higher arousal-index, as well as a higher total amount of arousals [23]. Additionally, the frequency of apneas or hypopneas per hour sleep (apnea index, hypopnea index) is related to the arousal index.

Surprisingly, few studies have investigated respiratory arousals in terms of their microstructure [5]. Arousal microstructure could be a key to understanding respiratory events. The objective of this study has been to further investigate the role of RAs in OSA-patients. It has been investigated whether not only the number, but also the duration and intensity (microstructure) of RAs correlate with polysomnography-related disease severity in OSA patients. On the other hand, it has been tested whether every individual might have a specific, stereotype arousal pattern which is independent of OSA-severity and also stable in its microstructure (i.e., duration and intensity on EEG recordings).

## 2. Materials and Methods

Full-night polysomnography (PSG) recordings of 20 patients with sleep-disordered breathing (SDB) who had been studied between April 2015 und June 2016 were retrospectively visually analyzed: 9 female and 11 male; age 27–80 years with a BMI range of 20–40.5 kg/m^2^. Patient data contained age, sex, weight, height, body mass index (BMI), RDI, arousal index (AI) amount of respiratory arousals, total sleep time (TST), arousal duration and amplitude as well as leg movements, desaturations and the Epworth Sleepiness Scale (ESS) score. Patients with an established diagnosis of isolated or concomitant narcolepsy, hypersomnia, chronic fatigue syndrome, restless legs syndrome, and/or diurnal rhythm disorders and patients with an established diagnosis of a psychiatric or neurologic (peripheral or central) disorder were excluded from analysis. In addition, patients with systolic or diastolic heart failure, history of myocardial infarction, peripheral vascular disease with associated surgical vascular procedure (including stent placement), chronic obstructive pulmonary disease requiring standard medication and patients with a history of malignant diseases were excluded from the assessment as well. Finally, patients treated with benzodiazepines, GABA-receptor agonists or opiates were excluded from the analysis.

All individuals had undergone overnight PSG with recordings of electroencephalogram (EEG), electrooculogram, submental and bilateral pretibial electromyogram and electrocardiogram. Nasal airflow was measured via impact pressure through a nasal sensor in which pressure fluctuations of the breathed air stream were determined. Thoracic and abdominal excursions, oxyhaemoglobin saturation (pulse oximeter) and body position were simultaneously recorded. Snoring was recorded with a pre-laryngeally fixed microphone. The polysomnographic recordings were performed using the Alice-LE-Diagnostic Sleep System (Philips Healthcare/Respironics, Best, Netherlands; supplied by Loewenstein Medical, Bad Ems, Germany). Polysomnography was performed in a standard way between 10 pm and 6 am for each patient. In the morning following each sleep study night, sleep stages and sleep-related respiratory events were manually scored according to the American Academy of Sleep Medicine (AASM)-2012 guidelines [24]. A nasal airflow amplitude reduction greater than 90%, lasting for at least 10 s, was defined as apnea. Hypopnea was defined as an airflow reduction between 50% and 90% with an associated 3%-reduction of the blood oxygen saturation (SpO_2_). Further classification in obstructive, central or mixed respiratory apnea events was based on simultaneous evaluation of nasal airflow and thoracic as well as abdominal excursion. Based on the built-in software of the PSG-device, the RA number during each individual’s sleep period as well as the duration and amplitude of each RA were visually registered. RAs were detected in 30-s epochs and were scored compliant to the criteria of AASM 2007 [4]. For every arousal, the corresponding respiratory event was recorded, e.g., apnea, hypopnea or respiratory-effort-related-arousal (RERA). A respiratory event was scored as a RERA using the AASM 2012-criteria [24], namely when there was a sequence of breaths lasting at least 10 s characterized by increasing respiratory effort or by flattening of the inspiratory portion of the nasal pressure flow waveform leading to arousal from sleep. Additionally, the sequence of breaths should not meet criteria for an apnea or hypopnea. A total of 2600 respiratory arousals were analyzed. Intensity and duration of every RA was measured with the help of Alice LE software. The RA intensity was determined manually and visually, supported by the Alice PSG software, by measuring the EEG-amplitude of the arousal, defined as the distance of the highest and lowest point on the vertical axis of the EEG for each arousal. In this manner, we could provide values of intensity as a continuous variable. All respiratory arousals included in the present analysis were registered on the C3M2-EEG lead. Severity of OSA was defined based on the RDI. The definition of RDI was based on the recommendations of the AASM [4], namely RDI = AHI + RERA index. A possible correlation between each one of the RA microstructure variables and RA number during each individual’s sleep period and the RDI and Arousal-Index was tested using the Spearman’s rho coefficient. Additionally, we did an outlier analysis based on the probability distributions of the measured parameters for each group. Intraclass correlations (ICC) for both RA duration and RA intensity were calculated. Moreover, we did a group calculation of the RA duration and RA amplitude separately for apneas and hypopneas. Full-night PSG was performed during two consecutive nights in the sleep lab. Only the second night of PSG was used for our investigations in order to minimize “first-night-effects” [25]. This was a retrospective review of data found in the patients’ charts and therefore permission had been provided by the local Institutional Review Board to use this data for research purposes, provided that patient confidentiality would be strictly respected (Nr. 2018-13942). The research presented in this manuscript has been conducted according to the principles of the Declaration of Helsinki.

### Analysis of a Patient-Individual Arousal Pattern

To obtain the RA pattern of each individual patient, we analyzed the arousal duration and the arousal intensity, which were measured manually using the Alice-PSG-software on the PSG chart. Independent factors of respiratory arousals, like oxygen saturation and sleep stage as an expression of EEG-activity, were also considered in the analysis. The probability distributions of the two parameters for each patient were plotted as a histogram and interpolated with a kernel (window), which fits the distribution. The number of bins to look at the distribution was selected to be five for both of the parameters and all the patients in order for them to be comparable over the whole group of patients. For data sets with a non-Gaussian distribution and not equal sample sizes we used the Bayesian posterior distribution analyses in order to identify the difference between each individual patient. This analysis provides complete distributions of credible values for means and their differences [26]. In particular, in testing individual differences, we opted for pair-wise comparisons between each patient to all other patients. From the analyzed 20 patients we built 190 pair-wise comparisons for each parameter separately. We previously successfully applied this type of analysis for classification and effect size estimation [27].

## 3. Results

The median age was 51.95 years with a standard deviation of ±13.80 years. RDI ranged in the examined individuals from 5.6 to 130.4/h and the AI ranged between 11.3/h and 64.5/h, with a mean value of 17.4/h. The amount of RAs varied between 46 and 402 per night. The median amount of RAs was 100.5 per night. The mean total sleep time was 363.75 min, with a standard deviation ±57.4 min. BMI ranged between 20 and 40.5 kg/m^2^ (average 29.5 kg/m^2^). Table 1 summarizes the patients’ characteristics and demographical data.

The measured data on intensity and duration of every arousal was recorded and a median duration and intensity (amplitude) of RAs was calculated for each patient (see Table 1).

### 3.1. Correlation Analysis of Different Polysomnographic Parameters with Arousal Duration and Amplitude

**Figure 1:**
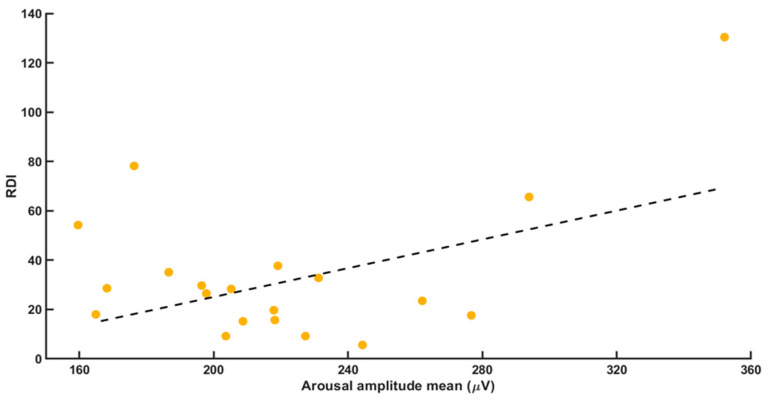
The correlation between the variables mean arousal amplitude and the RDI. We found a positive correlation with *r* = 0.446 and *p* = 0.038.

**Figure 2:**
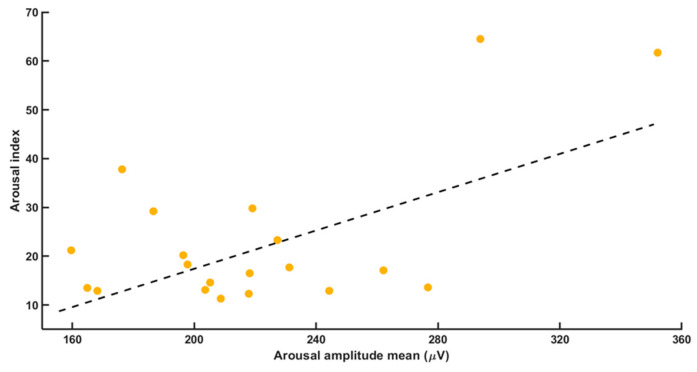
The correlation between the variables mean arousal amplitude and the RDI. We found a positive correlation with *r* = 0.446 and *p* = 0.038.

Correlation calculations showed a strong positive correlation between mean (average) RA intensity and both RDI (*r* = 0.446; *p* = 0.0038) and arousal index (*r* = 0.580; *p* = 0.007, Figure 1 and Figure 2).

Subinvestigations contained the analysis of intensity and duration of RAs in relation to two subgroups, namely in patients with RDI <30/h (moderate and mild OSA) and patients with RDI ≥30/h (severe OSA).

Interestingly, neither median duration nor median intensity differs significantly between the two RDI groups (Mann-Whitney-U-Test for duration *p* = 0.81; for intensity *p* = 0.751). The moderate and mild OSA subgroup showed medium respiratory arousal duration of 7.5 s and the severe OSA subgroup had medium arousal duration of 8.0 s. Mean arousal intensity was 203.5 µV in the moderate and mild OSA group compared to 206.5 µV in the severe OSA group.

As suspected and proven in various studies before [23], there was a strong positive correlation between arousal index and RDI: the respective Spearman’s Rho coefficient was *r* = 0.846 with *p* < 0.001. Correlation analysis of RA duration and RDI showed a weak negative correlation with a coefficient of *r* = −0.250 and *p* = 0.287. This underlines that the length of an arousal does not correlate with disease severity. Correlation of the intensity of the arousals with RDI was relatively weak, with a coefficient of *r* = 0.346 and *p* = 0.0483. No correlation could be found either between arousal index and mean RA duration (*r* = −0.084; *p* = 0.723) or between mean RA duration and RA intensity (*r* = 0.098 and *p* = 0.678). ESS showed no correlation to the arousal index (*r* = −0.038 and *p* = 0.87). Regarding the arousal intensity, ESS showed no correlation (*r* = −0.119 and *p* = 0.616).

**Figure 3:**
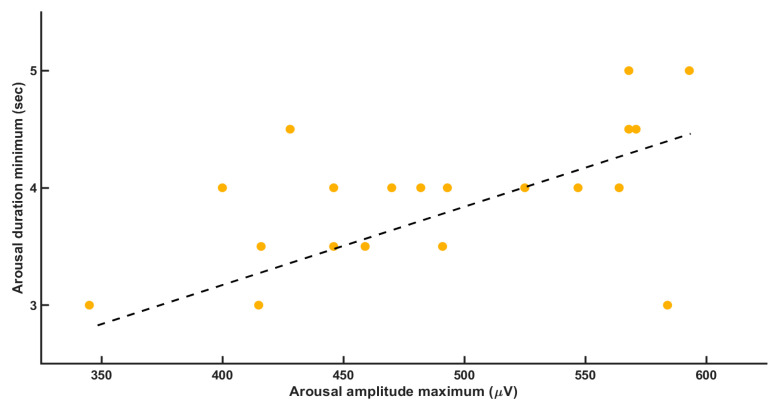
It shows the correlation between the variables arousal amplitude maximum and the arousal duration. We found a positive correlation with *r* = 0.534 and *p* = 0.0015.

We also found a positive correlation between minimal arousal duration and maximal arousal intensity (*r* = 0.534; *p* = 0.0015, Figure 3). Neither RA intensity nor RA duration correlated with the BMI (body mass index).

**Figure 4:**
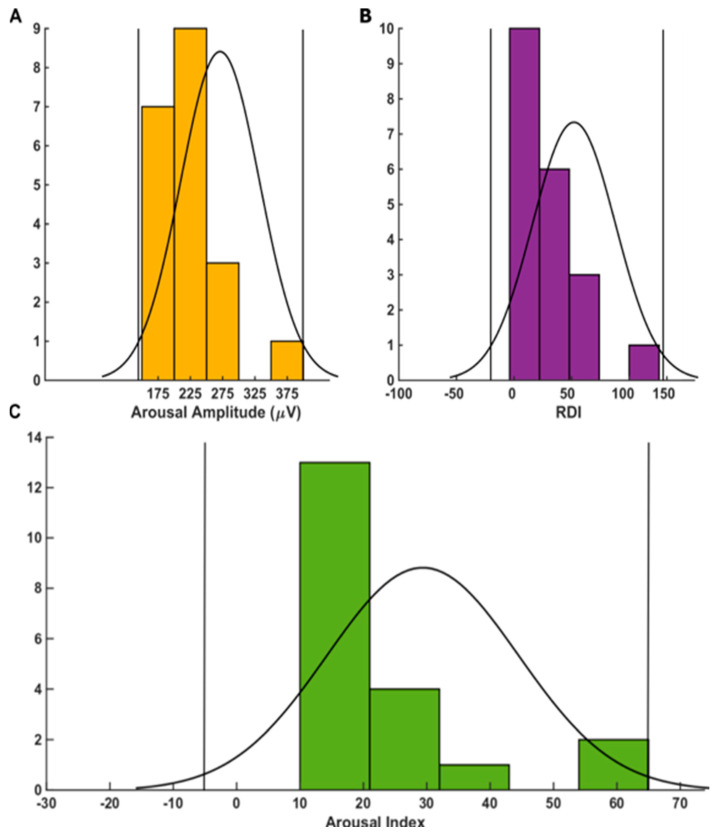
Outlier analyses based on the probability distributions of the measured parameters for each group. We set a 95% threshold to determine the outlier in the analyses. The parameters are arousal mean amplitude in (**A**), followed by RDI in (**B**), and the arousal index in (**C**).

Neither age nor sex correlated with either the average arousal intensity or with the average arousal duration, according to our analysis. The outlier analyses based on the probability distributions of the measured parameters for each group were plotted as a histogram and interpolated with a kernel (window) which fits the distribution. We found that values that would intuitively appear as outliers were actually not outliers in the distribution (Figure 4).

**Figure 5:**
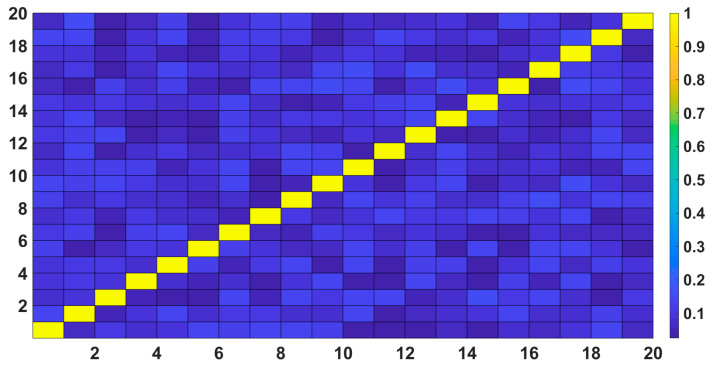
The plots below show the between-subjects ICC as a correlation matrix; except the diagonal all the other values were below (0.25) and was not significant (all *p* > 0.05).

**Figure 6:**
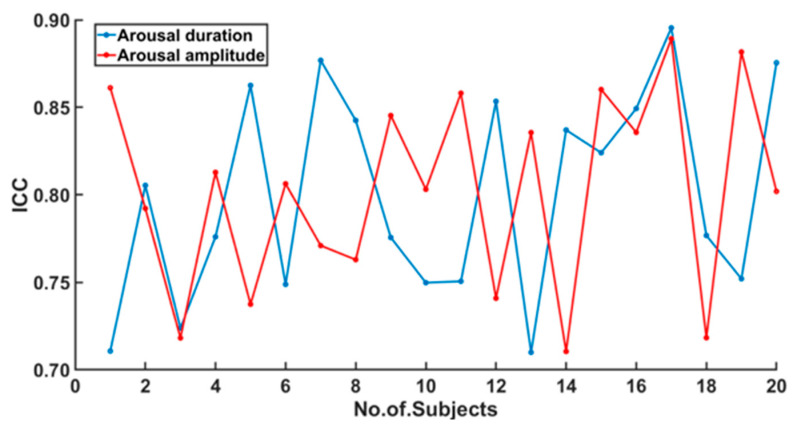
The within-subjects ICC values for each subject were plotted separately for the two parameters arousal amplitude (red) and arousal duration (blue). The ICC values were all above 0.7 and showed a significant within subject correlation (all *p* < 0.05).

The between-subjects ICC values for both RA duration and intensity were below 0.25 and were not significant (all *p* > 0.05). On the other hand, the within-subjects ICC values were all above 0.7 and significant (all *p* < 0.05) (Figure 5 and Figure 6).

In addition, we performed a separate detailed analysis of the RA intensity and duration in each sleep stage (NREM1, NREM2, NREM3, REM): we found no significant correlations between age or sex and either average arousal duration or average arousal intensity in any of the four different sleep stages. Of note, we did not find any significant correlations between RDI or arousal index, neither average arousal duration nor average arousal intensity, in any of the four different sleep stages. In contrast, we found significant correlations between average RA intensity in NREM2 (non-REM2) sleep stage and both total oxygen desaturation index (tODI; *r* = 0.578, *p* = 0.0075) and non-REM-specific ODI (*r* = 0.586, *p* = 0.0065). A similar correlation was found in NREM3 sleep stage for average arousal intensity and both tODI (*r* = 0.449, *p* = 0.046) as well as non-REM-specific ODI (*r* = 0.454, *p* = 0.044). Notably, a much more significant correlation between average arousal intensity in REM sleep stage and total oxygen desaturation index (*r* = 0.739, *p* = 0.0001) was found.

### 3.2. Apnea- and Hypopnea-Specific Analysis

**Figure 7:**
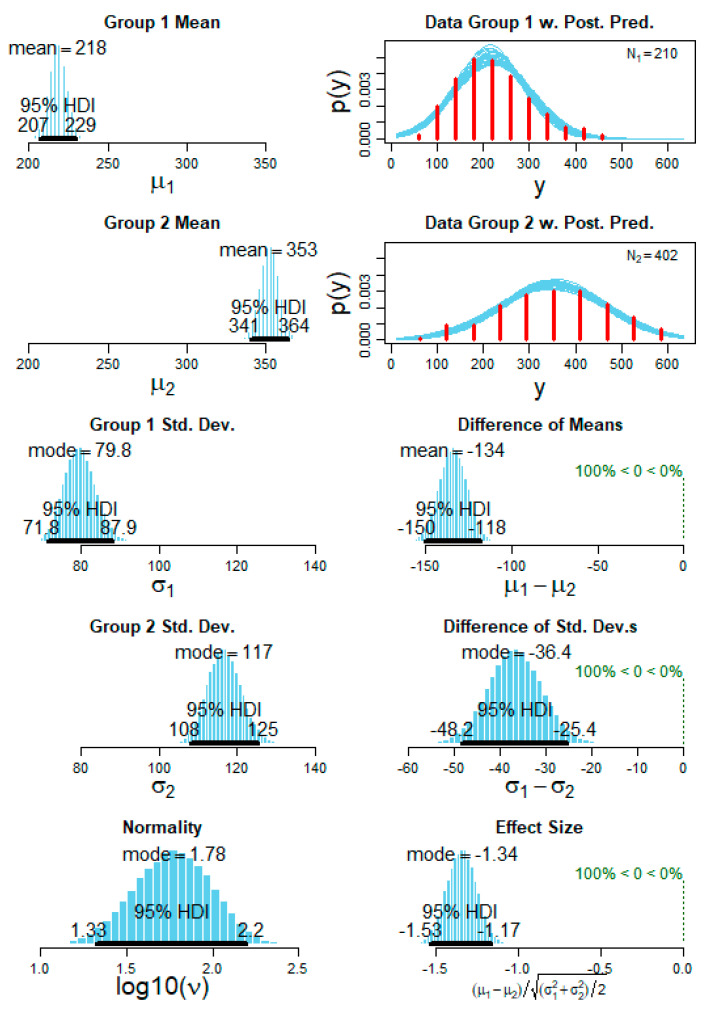
Calculation of the arousal duration and arousal amplitude separately based on the apnea and hypopnea. For the arousal duration we did not find any significant difference at the group level. However, the arousal amplitude (i.e., intensity) was significantly (*p* = 0.00135) higher in hypopnea-related arousals.

For the RA duration, we did not find any significant difference at the group level. However, the RA intensity was significantly (*p* = 0.00135) higher in hypopnea-related arousals, as shown in Figure 7.

### 3.3. Analysis of Individual Arousal Patterns in Each Patient

**Figure 8:**
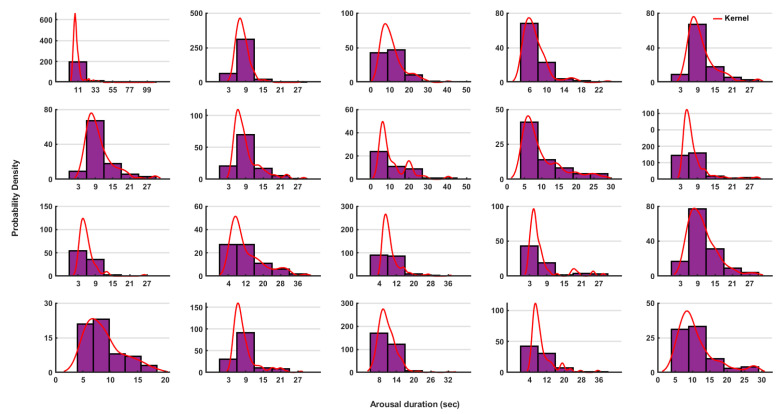
The probability distributions of the arousal duration (in seconds) are shown for each patient separately. The histogram depicted in violet color and the interpolated kernels are shown in red color. The respective values showed a log-normal distribution.

In the case of the RA duration, we found a clear individual pattern for each patient (Figure 8). Furthermore, in most patients, we found a log normal distribution. Using the Bayesian statistics, we were able to differentiate between each pair of patients with an accuracy range of 80–100%. An example of the comparison between two patients is shown in Figure 8.

**Figure 9:**
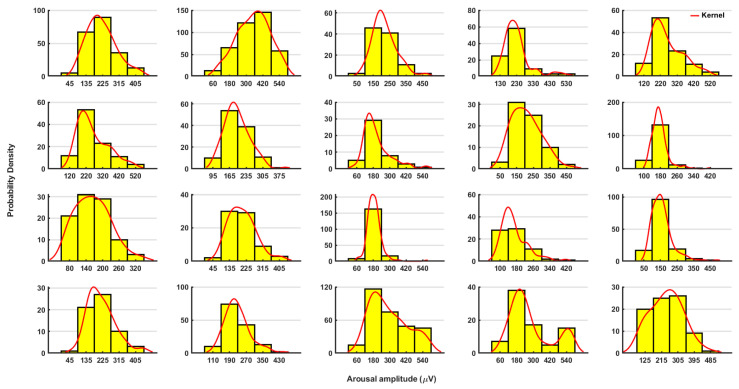
The probability distribution of the arousal amplitude (in µV) is shown for each patient separately. The histogram depicted in yellow color and the interpolated kernels are shown in red color. The patients showed a normal (Gaussian) distribution. The number of bins was chosen as *n* = 5.

**Figure 10:**
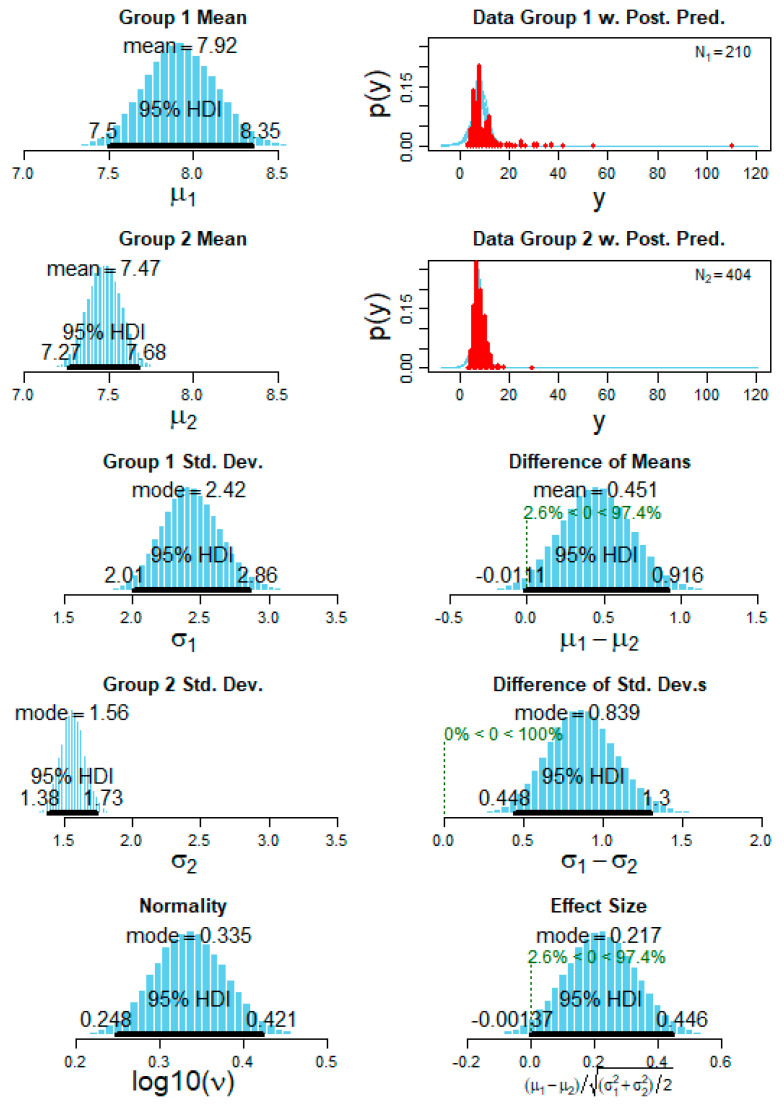
A representative example of the Bayesian statistics of the posterior predictive distribution (PPD) for the variable arousal amplitude. The first column (top to bottom) shows the subject 1 mean PPD, subject 2 mean PPD followed by standard deviation of each subject and finally the normality PPD for the datasets. The second column (top to bottom) shows the subject 1 raw values distribution with the interpolated kernel (*N*1 = 210 arousals for subject 1) and the subject 2 raw values distribution (*N*2 = 402 arousals for subject 1) followed by the difference of the means and standard deviation and finally checking the effect size for the comparison of the two subjects’ data. The difference of means shows a clear distinction of (100%) between the two subjects and also the standard deviation showed a clear distinction with (100%). For all the PPD graphs, the 95% highest density interval (HDI) is shown as dark black lines.

On the other hand, regarding the RA intensity (amplitude) a normal (Gaussian) distribution was found in most patients (Figure 9). The Bayesian statistics for the parameter RA intensity (amplitude) showed significant differences between each pair of patients with an accuracy range of 85–100%. The comparison for the RA intensity between the same patients as in Figure 1 is now shown as an example in Figure 10.

## 4. Discussion

In summary, our investigations suggest that the RA intensity is positively correlated with both RDI and arousal index. As a result, RA intensity is related (while RA duration is not) to OSA disease severity and arousal frequency during sleep in OSA patients. No sleep stage-specific correlations of arousal microstructure with age, sex, RDI or AI were identified. Although duration of apnea-related RAs did not differ from duration of hypopnea-related RAs, arousal intensity was significantly higher in hypopneas than in apneas. Additionally, we found evidence that the microstructure of a RA might be patient-specific. This implies that every individual patient with OSA has a cortical or sub-cortical neural arousal-associated pattern generator, which reacts to an obstructive respiratory event with a stimulus and a specific signature in terms of duration and intensity, like a distinct pattern, in order to ensure ventilation during sleep. Alternatively, in some individuals with OSA, this neural pattern generator may pro-actively induce respiratory arousals preceding the obstructive events in OSA patients.

The number of patients, whose polysomnographic recordings were analyzed, can be discussed, since only 20 patients were analyzed. In such a small cohort, results could be easily influenced by confounders. Patients with comorbidities, like insomnia, that are known to be very common in OSA patients and appear to have a great influence on sleep quality, ESS-scores and effectiveness of OSA-treatment, were excluded [28]. Other studies on arousals in OSA patients included a considerably higher number of participants [1,23,29]. Nonetheless, in the present study a rather great number of RAs per patient was analyzed, resulting in a total of 2600 RA for analysis. Since to our knowledge there are no studies with a similar methodology on respiratory arousals to date, a comparison may be lacking. Additionally, artifacts of EEG-signals could have influenced and hence confounded the measurement of the exact arousal intensity and/or duration. These overlays might have been caused by the detachment of electrodes during the night or incorrect measurement of the electrical impedances preceding the polysomnography. Besides, only patients with OSA were analyzed, and hence there was no control group for comparison. To further examine the findings presented in this manuscript, recordings from a larger patient cohort are needed. Moreover, to reduce any confounding impact of aging processes on the data and results, a more homogeneous cohort by age of the OSA patients should be investigated in future studies.

In several studies, a weak correlation between arousal index and ESS was observed [23,30]. In the present study, we dealt with the correlation of the microstructural arousal parameters with the subjective malady displayed by the ESS questionnaire, which was found to be insignificant. There are certainly several reasons for the lack of correlation between the ESS and the objective parameters of polysomnography in our data. The perception of severity differs between individuals. Besides, patients often only realize the severity of their previous restrictions after successful treatment [31].

Explanations of the genesis and usefulness of respiratory arousals are ambivalent. They seem to terminate or attenuate severe respiratory events and their consequences by interrupting the apnea/hypoxic event [8]. However, physiologic changes that accompany an arousal are believed to be harmful and to lead to the maintenance or even worsening of unstable breathing [1]. Moreover, in some patients, RAs may actually lead to an exacerbation of the OSA disease [1,3]. One theory is that if arousals did not occur in an apneic or hypopneic event, the episode would last longer and the apnea-hypopnea-index (AHI) would eventually decrease. RA may thus indeed lead to a higher degree of sleep fragmentation and prevent the patients from entering to deeper sleep stages, where obstructive events are much less frequent [1].

It has been suggested that respiratory arousals trigger more respiratory events that are terminated by the arousal itself [3]. An arousal is believed to be followed by “over-breathing”, then the patient returns to sleep and there follow phases of “under-breathing” with obstructions of the upper airway, which are terminated again by an arousal with a consecutive “over-breathing” [3]. The connection between arousal index and RDI is undeniable. In a study by Gonçalves, that included and examined 135 men with a mean RDI of 48.7/h ± 26.8, a strong correlation between RDI and arousal index was observed (*r* = 0.783; *p* = 0.001), similar to our findings [23].

Amatoury et al. investigated whether the intensity of an arousal depends on the strength of the preceding respiratory stimulus as well as its role on post-arousal ventilator and pharyngeal muscle responses. In their study, average arousal intensity was not related to the strength of the respiratory stimulus, but there was a positive correlation to the arousal duration and the latency of the arousal after the stimulus, the so-called time to arousal, as well as a negative correlation to the BMI [32]. The authors concluded that the arousal intensity is a distinct pathophysiological trait in obstructive sleep apnea, which supports our hypothesis of a cortical (or subcortical) arousal patient-specific pattern. Besides, in this study, the muscle responses to arousals increased with increasing arousal intensity. The authors thus suggested that higher arousal intensity may lead to respiratory instability [32]. Muscle response was not considered in our study. In this interventional study by Amatoury et al., a period of 3 min of continuous positive airway pressure (cPAP) withdrawal was used, in order to induce RA. The patients in which arousals were analyzed were thus not naïve to therapy and only some periods of the night with study-induced sleep interruptions were analyzed. In contrast, our study is purely observational, and involves full-night polysomnographic data including all respiratory arousals of patients with a first diagnosis of OSA without any prior specific therapy for OSA. Additionally, in the Amatoury et al. study, arousal intensity was divided into categorical groups. Intensity was measured and scaled between 0 and 9 (using a validated automated wavelet transformation method as previously described by Azarbarzin et al. [7], and arousals were categorized into low (scale ≤5) and high (>5) intensities. In contrast to our study, Amatoury et al. used wavelet transform analysis as a system for quantification of arousal intensity. Therefore, a direct comparison of the results of these authors [7] and ours is not possible. A time-frequency analysis would have also been an appropriate method to estimate arousal intensity. The lack of this analysis is a limitation of our study and can be used as a starting point for upcoming analysis. Younes et al. [6] used a very subjective method to provide categorical classification of the arousal intensity. Our approach with an estimation in this detailed and objective way handles arousal intensity as a continuous variable and is therefore more precise. On the other hand, it is more time-consuming. Therefore, the microstructure (more specifically, the RA intensity) was not analyzed as a continuous variable by Amatoury et al. A control group of 17 healthy individuals (apnea hypopnea index, AHI <10/h) was also analyzed [32]. In our study, no control group was included, and this is the reason correlation coefficients were used to study possible association between the study parameters.

Individual RA intensity may therefore be influenced by OSA severity and the number of RA is quite strongly correlated with OSA severity, although the RA duration is not associated with OSA severity. Given that both RA intensity and duration show an individual patient-specific pattern, it may be that central nervous system RA pattern generation is quite robust in each individual and that the intensity of this pattern may be positively associated with the frequency of RA pattern generation, as depicted by the arousal index, or with the frequency of obstructive respiratory events (as depicted by the RDI) during sleep. Still, these results must be interpreted with caution, since the participants number was low, and only a single night of polysomnography was analyzed.

Our finding of very high within-subject intraclass correlations for RA duration and intensity (Figure 5 and Figure 6) further supports the hypothesis of individual pattern of RA duration and intensity. Additionally, the observation that RA intensity was significantly higher in hypopneic than in apneic events may be causally associated with a mechanism promoting less respiratory distress (the one associated with hypopneas) compared to the more severe respiratory distress associated with apneas.

A study by Huang et al., who analyzed respiratory events in the sense of oxygen desaturation and EEG-changes, showed that the proportion of respiratory events followed by a stabilization of breathing, instead of unstable breathing with or without an arousal, increase as sleep deepens [31]. One could conclude that the arousal structure in slow wave sleep would be different. However, in our study, we did not find any significant differences in arousal structure throughout the sleep stages.

Besides, Huang et al. showed that respiratory events that lead to an interruption of breathing are more severe in terms of EEG-changes and oxygen saturation drop than events without breathing interruptions [31]. The assumption that arousal microstructure, in particular arousal intensity, could be associated with peripheral blood oxygen saturation was also supported by our findings: arousal intensity in NREM2, NREM3 and REM was significantly correlated with total ODI.

Respiration is mainly controlled by the autonomic nervous system (ANS), whereas respiratory arousals are a central nervous system’s (CNS) response. The ANS regulates the cardiovascular function, while respiratory events influence the ANS [33]. Cortical and cardiac oscillations reflect the communication of CNS and ANS during sleep [34], called the ANS-CNS coupling. In their study, Liang et al. showed that the combination of respiratory events and arousals has a greater effect on heart-rate-variability, as an expression of ANS activity, than both events individually [35]. Moreover, the extent of their effect on the ANS depends on the sleep stage and thus on the CNS-activity. Therefore, the coupling between ANS and CNS seems to play an important role in sleep microstructure. Our study did not include the heart rate variability as a direct expression of ANS function. This could be the subject of further studies in order to find the effect of ANS response to abnormal breathing on individual arousal patterns.

In animal studies, evidence suggests that the two brain subcortical regions that promote waking or wake-associated phenomena related to arousal are the basal forebrain and the parabrachial region. The parabrachial complex (PBC) is a visceral sensory nucleus in the rostral pons, which projects to the cerebral cortex and has been studied extensively in rats [36]. The PBC is well-positioned to access relevant data to determine respiratory distress and cause an arousal, since it is a key component of the central respiratory and the arousal network [37,38]. Furthermore, serotonin and glutamatergic signaling seem to be required to cause an arousal [3]. Infants suffering sudden infant death syndrome (SIDS) have an arousal deficit as a contributing factor to SIDS. Their brainstems are deficient in serotonin (5-HT) and have decreased binding to 5-HT receptors [39,40]. Eliminating medullary 5-HT neurons leads to a delay of arousal and decreases the respiratory response to hypoxia in rats [39]. It may be that, depending on OSA severity, the serotoninergic system fine-tunes the activity of the background cortical (or subcortical) RA neural pattern generator by modulating its firing intensity.

In this work, the microstructure of arousals, and not the exact timing of an arousal, was analyzed. Following Sun et al., the structure of certain EEG-parameters, like the K-complex that occurs during respiratory events, is significantly different from the structure that occurs right after respiratory events [40]. Respiratory arousals could have the same or similar characteristics. This should be the subject of further studies.

Indeed, an additional method to study arousal microstructure is by means of the arousal morphology. The cyclic alternating pattern (CAP) consists of transient arousals (phase A) that periodically interrupt the tonic theta/delta activities of NREM sleep (phase B). It is possible to distinguish three subtypes of A phases corresponding to different levels of arousal power: A1 (high-voltage slow waves—EEG synchrony), A3 (dominated by low-amplitude fast rhythms—EEG desynchronization) and A2 (a mixture of A1 and A3) [41]. Sleep fragmentation in OSA patients has been associated with a significant enhancement of CAP and of the A phases with longer and more desynchronized EEG patterns (especially A3). The great majority of respiratory pauses (96% in NREM and 80% in REM sleep) were coupled with CAP [42]. CAP correlated significantly with the arousal index during NREM sleep. CAP rate, especially the A2+A3 index, was inversely related to self-reported quality of sleep, independent of age and sleep disturbance measures [43]. The CAP-method posts a different approach to arousal analysis. Nevertheless, it has been shown that it can be used to assess and distinguish the severity of OSA and its symptoms. For example, differences in CAP rates, duration and CAP-cycles could be shown in patients with daytime sleepiness by Korkmaz et al. [44] and Karimzadeh et al. found an increase in CAP rates among OSA subjects compared to healthy controls [45].

### Limitations of the Study

In summary, the limitations of our study include the limited number of patients and the lack of a control group without OSA, which would have allowed a comparison to healthy subjects. Furthermore, our selection represented a very inhomogeneous selection of patients. A more homogeneous cohort might have led to a reduction of confounding impacts.

Furthermore, we measured arousal duration and amplitude. A time-frequency analysis would also have been appropriate and an established method to determine arousal intensity. Because we analyzed the patients’ arousal data from only one night of polysomnography, the data must be evaluated with caution because one night is certainly not representative of the complete sleep structure of the patients.

The mechanisms through which airway obstruction causes arousals in humans are still poorly understood. Multiple contributing stimuli are believed to cause arousal. Since in many cases arousals are unnecessary, and can even worsen the severity of OSA, it would be interesting to study whether therapeutic interventions may change one or more of the RA microstructure parameters or the number of RA during sleep. A study of the correlation between these changes and clinical outcomes might also be interesting. Most importantly, a priority in the research agenda would be to localize more precisely the anatomic central nervous system structures involved in RA generation in humans.

## Figures and Tables

**Table 1:** Demographic data of the study participants (RDI: respiratory disturbance index, BMI: body mass index, ESS: Epworth sleepiness scale).

Demographic Data	Median (SD)	Range
Age	51.95 years (±13.809)	27–80 years
Amount of respiratory arousals	100.5/night	46–402/night
Arousal index	17.4/h (±15.329)	11.3–64.5/h
BMI	29.5 kg/m^2^ (±5.456)	20–40.5 kg/m^2^
ESS	12 (±5.719) points	2–20 points
Mean RA amplitude (range)		150–229.5 µV
Mean RA duration (range)		5–10.25 s
Proportion of women	45%	
RDI	34.04/h (±29.353)	5.6–130.4/h
Total sleep time	363.75 min (±57.4)	188.5–423.5 min

## Data Availability

The data presented in this study are available on request from the corresponding author. The data are not publicly available due to privacy.
